# Clinical Findings in a Multicenter MRI Study of Mild TBI

**DOI:** 10.3389/fneur.2018.00836

**Published:** 2018-10-23

**Authors:** Teena Shetty, Joseph T. Nguyen, Taylor Cogsil, Apostolos John Tsiouris, Sumit N. Niogi, Esther U. Kim, Aashka Dalal, Kristin Halvorsen, Kelianne Cummings, Tianhao Zhang, Joseph C. Masdeu, Pratik Mukherjee, Luca Marinelli

**Affiliations:** ^1^Department of Neurology, Hospital for Special Surgery, New York, NY, United States; ^2^Biostatistics Core, Hospital for Special Surgery, New York, NY, United States; ^3^Department of Radiology, Weill Cornell Medicine, New York, NY, United States; ^4^New York Medical College, Valhalla, NY, United States; ^5^GE Global Research Healthcare, Waukesha, WI, United States; ^6^SUNY Downstate Medical Center, Brooklyn, NY, United States; ^7^Department of Neurology, Houston Methodist Hospital Houston, TX, United States; ^8^Department of Radiology, University of California, San Francisco, San Francisco, CA, United States; ^9^GE Global Research Center, Niskayuna, NY, United States

**Keywords:** mild traumatic brain injury, age, gender, neuropsychological assessments, concussion, imaging, white matter hyperintensities, risk factors

## Abstract

**Background:** Uncertainty continues to surround mild traumatic brain injury (mTBI) diagnosis, symptoms, prognosis, and outcome due in part to a lack of objective biomarkers of injury and recovery. As mTBI gains recognition as a serious public health epidemic, there is need to identify risk factors, diagnostic tools, and imaging biomarkers to help guide diagnosis and management.

**Methods:** One hundred and eleven patients (15–50 years old) were enrolled acutely after mTBI and followed with up to four standardized serial assessments over 3 months. Each encounter included a clinical exam, neuropsychological assessment, and magnetic resonance imaging (MRI). Chi-square and linear mixed models were used to assess changes over time and determine potential biomarkers of mTBI severity and outcome.

**Results:** The symptoms most frequently endorsed after mTBI were headache (91%), not feeling right (89%), fatigue (86%), and feeling slowed down (84%). Of the 104 mTBI patients with a processed MRI scan, 28 (27%) subjects had white matter changes which were deemed unrelated to age, and 26 of these findings were deemed unrelated to acute trauma. Of the neuropsychological assessments tested, 5- and 6-Digit Backward Recall, the modified Balance Error Scoring System (BESS), and Immediate 5-Word Recall significantly improved longitudinally in mTBI subjects and differentiated between mTBI subjects and controls. Female sex was found to increase symptom severity scores (SSS) at every time point. Age ≥ 25 years was correlated with increased SSS. Subjects aged ≥ 25 also did not improve longitudinally on 5-Digit Backward Recall, Immediate 5-Word Recall, or Single-Leg Stance of the BESS, whereas subjects < 25 years improved significantly. Patients who reported personal history of depression, anxiety, or other psychiatric disorder had higher SSS at each time point.

**Conclusions:** The results of this study show that 5- and 6-Digit Backward Recall, the modified BESS, and Immediate 5-Word Recall should be considered useful in demonstrating cognitive and vestibular improvement during the mTBI recovery process. Clinicians should take female sex, older age, and history of psychiatric disorder into account when managing mTBI patients. Further study is necessary to determine the true prevalence of white matter changes in people with mTBI.

## Introduction

Mild traumatic brain injury (mTBI) is defined as a traumatically induced physiological disruption of brain function (1). Although mTBI accounts for at least 75% of traumatic brain injuries and imposes an excessive societal burden (2, 3), mTBI diagnosis continues to lack objective clinical and imaging biomarkers. As of now, the best marker for severity and recovery is a subjective assessment of acute symptom burden (4). Uncertainty continues to surround mTBI diagnosis, symptoms, prognosis, and outcome for physicians and patients as reliable biomarkers remain elusive. As injury rates increase and mTBI becomes a serious public health epidemic (5), there is an increasing role for identification of potential imaging biomarkers, specific neuropsychological assessments, and validated risk factors to help guide prognoses and return to play decisions.

Given the current subjective nature of symptom burden assessment, there is a role for neuropsychological assessments in evaluating the cognitive impairment of patients after injury. The sport concussion assessment tool (SCAT) has been demonstrated as an effective tool to differentiate between mTBI subjects and controls in non-athlete populations and is widely used in mTBI studies (6–8). Tests of memory, balance, and cognition are incorporated into the SCAT (9, 10), but research has not demonstrated their effectiveness as longitudinal assessments (11). Separately, 3-word recall is commonly employed in patients with mTBI to assess memory function (12). This test is usually normal and is probably inadequate for assessing these patients.

The risk factors for mTBI severity are debated in the literature. Demographic factors commonly explored include sex, age, previous concussions, learning disability, psychiatric history, and migraine/headache history (13). Although each of these preinjury characteristics has been studied in numerous protocols, a consensus has not been reached. Further research is needed to establish the risk factors for mTBI severity so that they may be incorporated into clinical care.

Moreover, routine imaging techniques are limited in their value of serving as biomarkers of severity or prognosis in the mTBI population, and the extent of incidental magnetic resonance imaging (MRI) findings in mTBI patients also remains unclear. Conventional structural MR imaging is felt to be limited in its yield of disease severity or prognosis. Further research is necessary to investigate the anatomical characteristics of the mTBI population that present to medical attention. Better characterization of the specific abnormalities in anatomic imaging in this population is necessary.

The aim of this study was to incorporate patient history, clinical exams, imaging, and multiple neurological assessments into a prospective longitudinal study of patients presenting with an acute mild traumatic brain injury to provide guidance for hypothesis generation and future study design of mTBI research. Traditional neuropsychological assessments were developed to further attempt to detect abnormalities in patients with mTBI. Although MR imaging is not routinely performed for acute mTBI, recent advances in MRI based techniques have allowed researchers to incorporate imaging into mTBI trials. This study specifically investigated the presence of white matter hyperintensities on structural imaging. This combination of assessments and time points provided a more comprehensive and detailed assessment of symptoms and outcomes of mTBI patients than found in previous studies. This allows for identification of previously elusive potential risk factors which may influence outcome measures for mTBI populations.

## Methods

This study was conducted at three sites: Hospital for Special Surgery (HSS), University of California San Francisco (UCSF), and Houston Methodist Hospital (HM). All procedures were compliant with the Health Insurance Portability and Accountability Act (HIPAA) and were approved by each institution's Institutional Review Board (IRB). All participants or guardians granted written informed consent to participate. A uniform protocol was followed at each respective site to ensure standardization of data.

### Study population

Inclusion criteria were age between 15 and 50 years and a clinical diagnosis of mTBI with Glasgow Coma Scale (GCS) ≥ 13. mTBI was defined as a low-velocity injury that results in clinical symptoms but is not necessarily related to a pathological injury, in accordance with both the Zurich Guidelines and those of the American Congress of Rehabilitation Medicine (1, 14). Mechanism of injury was not an inclusion or exclusion criterion, as our population was not restricted to sport-related concussion. Patients were excluded for prior moderate or severe brain trauma, mTBI within the year, neurological or neuropsychological abnormalities, or medication that may influence assessments (detailed exclusion criteria in Appendix e-1). Patients were enrolled at either Encounter 1 (E1), within 72 h, or Encounter 2 (E2), 5–10 days post-trauma. They returned for a maximum of 4 encounters over 3 months. Encounter 3 (E3) occurred at 15–29 days and Encounter 4 (E4) at 83–97 days. All time points were chosen based on previous literature. E1 and E2 allowed us to capture the acute impairments, and E2 was used to capture the majority of individuals who are reported to recover within 10 days (15). However, studies show that comorbidities may prolong recovery for several weeks, which is captured by E3 (16). Lastly, most studies suggest complete resolution of symptoms by 3 months, which is captured by E4 (17).

At enrollment, medical and family history were collected, including psychiatric disease, learning disability, previous mTBIs not within the last year, and migraines or other headache types. Principal investigators assessed migraine by physician diagnosis. At all visits, subjects underwent a clinical examination, neurological assessments, and a multimodal MRI. MRI scans were completed within 24 h after completion of clinical neurological evaluations. After study completion, date of patient recovery was determined for subjects enrolled at HSS.

Normal control subjects were recruited from a sample similar to the patients, e.g., friends or classmates of mTBI patients, and enrolled to assess the viability of differentiating clinical neurological and MR exams against mTBI patients. Control subjects shared the same exclusion criteria as the mTBI subjects, and were matched based on age, education level, sex, and handedness. Controls completed neurological assessments and multimodal MRI at an initial and follow-up visit between 7 and 90 days, with an average of 3 weeks between the initial and follow up encounters.

### Assessments

All subjects were administered a Composite General Symptoms Assessment (CGSA) at each encounter. The CGSA is a comprehensive tool that combines the Sports Concussion Assessment Tool 2 (SCAT2) with other established clinical tools, such as the Balance Error Scoring System (BESS) and the concussion graded symptoms checklist (GSC) (9, 18–20). The total symptom severity score (SSS) captured ranges from 0 to 132 with higher score indicating greater symptom severity. The CGSA also includes tests of specific cognitive domains found in the SCAT: immediate memory (5-Word Recall), concentration (Digits Backward), and balance (modified BESS) (10). A full list of assessments included in the CGSA can be found in Appendix e-2.

### Imaging

MR imaging was performed on 3T GE Signa MR750 (GE Healthcare, Waukesha, WI) scanners with a 32-channel brain radiofrequency coil (Nova Medical, Wilmington, MA). A comprehensive imaging protocol included high-resolution volumetric structural, susceptibility and diffusion weighted imaging as well as investigational pulse sequences (described in detail in Appendix e-3). The MR data was standardized by use of a uniform protocol, scanner manufacturer and model, radiofrequency (RF) coil, and sequences as well as centralized processing.

Expert board certified neuroradiologists (AJT, PM) performed the safety reads and interpreted the structural imaging. Patients were classified with white matter abnormalities if ≥5 objective, punctate white matter foci were present. If < 5 foci were present, the white matter was classified as abnormal if lesions were >3 mm or located in atypical locations. Patients with abnormal white matter changes underwent neurological follow-up with an attending neurologist (TS) and routine clinical MRI.

### Statistical analysis

In regard to age, the study population was analyzed in two groups: those aged ≥25 and those < 25 years. This dichotomization was chosen based on the age when most brain structures typically reach developmental maturity (21–23).

Statistical analysis included reporting of means and standard deviations for continuous variables and frequencies and percentages for discrete variables. Independent sample *t*-tests and chi-square analyses were utilized for group comparisons. Similar tests were used to compare outcomes between patients with and without white matter abnormalities. Evaluations of all outcomes were performed using generalized linear models. The modeling technique is robust enough to handle data that do and do not meet the assumption of normality. Additionally, the modeling technique also allowed for all observations from all responders at each time point to be analyzed using maximum likelihood estimations (MLE). By clustering the data into longitudinal clusters for each patient, the model controlled for the missing data at each time point. Parameter estimates were reported using maximum likelihood estimates with statistical significance at *p* < 0.05. Bonferroni corrections were performed for multiple comparisons.

MATLAB (Mathworks, Natick, MA) and SPSS version 23.0 (IBM Corp., Armonk, NY) were used for all analyses.

## Results

### Study population

111 mTBI patients were enrolled (86 at HSS, 14 at HM, 11 at UCSF) between February 2014 and June 2015, 48 at E1 and 63 at E2. The number of observations included in encounters 1 through 4 were 48, 99, 83, and 59, respectively. Of these subjects, 48 were women (44), mean age was 23.2 ± 9.0 years, and 35 had sustained at least one previous mTBI prior to the last year. A total of 34 controls (26 HSS, 6 UCSF, 2 HM) were also enrolled. Other than number of previous mTBIs, statistical testing did not reveal any significant demographic differences between mTBI and controls (Table 1). Cause of injury for mTBI patients was as follows: 22 subjects were involved in a motor vehicle accident (20%), 73 were injured playing a sport (66%), and 18 were injured by a fall or other accident (16%). In terms of mechanism of injury, 48 subjects experienced a direct impact of their head against an object (45%), 46 had a direct impact of a blow to the head (44%), 4 had a ground level fall (4%), 1 had a fall from height <1 meter (1%), and one experienced acceleration/deceleration (non-impact) (1%).

**Table 1:** Subject and control demographics.

**Variable**	***Non-TBI Total***	**Non-TBI Control**		***mTBI Total***	**mTBI Case**		***P-*value**
		***N***	**%**		***N***	**%**	
**Patient age (Mean, SD)**	34	25.3	7.7	111	23.2	9.0	0.227
Age < 25	34	17	50%	111	73	66%	0.097
Age 25+	34	17	50%	111	38	34%	
**SEX**							
Male	34	18	53%	110	62	56%	0.726
Female	34	16	47%	110	48	44%	
**LEVEL OF EDUCATION**							
Less than HS	34	9	26%	110	52	47%	0.071
HS or GED Graduate	34	1	3%	110	7	6%	
Some college	34	4	12%	110	16	15%	
College degree (Bachelors or Associates)	34	13	38%	110	25	23%	
Graduate degree (e.g., Masters, MD, JD, PhD)	34	7	21%	110	10	9%	
**ETHNICITY**							
Hispanic or Latino	34	4	12%	111	12	11%	0.770
Not Hispanic or Latino	34	27	79%	111	97	87%	
Not Reported	34	3	9%	111	2	2%	
**RACE**							
White	34	18	53%	111	80	72%	0.887
Non-white	34	7	21%	111	29	26%	
Not Reported	34	9	26%	111	2	2%	
**SMOKING STATUS**							
Never smoked/used tobacco products	34	31	91%	109	98	90%	0.828
Current or former smoker/user tobacco products	34	3	9%	109	11	10%	
**PREVIOUS MTBIS**							
None (0)	34	34	100%	110	75	68%	0.001
1	34	0	0%	110	18	16%	
2+	34	0	0%	110	17	15%	
**PERSONAL HX - CHRONIC HEADACHE/MIGRAINE**							
No	34	34	100%	111	106	95%	0.591
Yes	34	0	0%	111	5	5%	
**DOMINANT HAND**							
Left	34	4	12%	110	13	12%	>0.999
Right	34	30	88%	110	97	88%	

### Acute clinical and MRI findings

At enrollment, the most endorsed symptoms were headache (91%), not feeling right (89%), fatigue (86%), and feeling slowed down (84%). Control patients had an average SSS of 1.68 (SD: 7.14). At enrollment, mTBI subjects scored significantly worse than controls on 5- and 6-Digit Backward Recall (*p* = 0.003, *p* = 0.006, respectively), single-leg and tandem-leg stances of the BESS (*p* < 0.001 for both), and Immediate 5-Word Recall (*p* < 0.001).

Of the 104 mTBI patients with at least one processed MRI scan, 28 subjects (27%) had white matter changes, as seen on T2 FLAIR. Of these 28 subjects, 15 (54%) had white matter changes that were deemed abnormal for age as previously described. In 26 patients (93%), these white matter findings were determined unrelated to the acute head trauma, as there were no additional supportive findings (restricted diffusion or microhemorrhages) to suggest acuity, and the findings were stable across all encounters. One patient had acute shear injury of the corpus callosum on imaging. Another patient demonstrated a focal subarachnoid hemorrhage along the right trochlear nerve with clinical findings of right trochlear nerve palsy. In the 32 controls, 9 were found to have had changes in white matter (28%), of which 3 (33%) were deemed abnormal for age. The frequency of change in white matter was not significantly different between mTBI and control populations (*p* = 0.840). When comparing neurological exams between patients with and without white matter abnormalities, no significant differences were found. White matter abnormalities were not associated with previous mTBIs or history of participation in contact sports. Additionally, there were no associations between personal history of migraine and white matter hyperintensities.

### Longitudinal findings

The average length of days since injury for each encounter was (E1) 1.98 ± 0.99, (E2) 7.82 ± 1.93, (E3) 22.11 ± 4.87, and (E4) 91.61 ± 5.53. For mTBI subjects, the average SSS for each encounter were E1 = 32.5, E2 = 32.6, E3 = 16.8, and E4 = 7.6. A significant difference is seen from E2 to E3 (*p* < 0.001) and E3 to E4 (*p* = 0.024).

**Figure 1:**
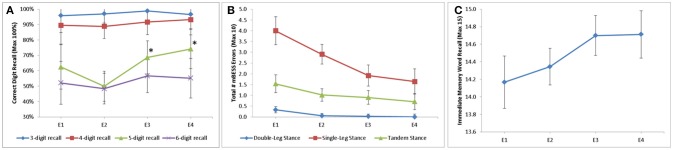
Longitudinal change in assessment scores stratified by **(A)** digit recall; **(B)** modified BESS; and **(C)** Immediate word recall. 5-Digit Backward Recall significant from E2 to E3 (50 to 69%, *p* = 0.009) and E2 to E4 (50 to 74%, *p* = 0.002). Double-Leg Stance, Single-Leg Stance, and Tandem-Leg stance of BESS all significant longitudinally (*p* = 0.003, *p* < 0.001, *p* = 0.028, respectively). Immediate 5 word recall statistically significant longitudinally (*p* = 0.007). ^*^p ≥ 0.05.

Of the neurological assessments, 5-Digit Backward Recall, the modified BESS, and Immediate 5-Word Recall saw statistically significant improvement over time in the mTBI population (Figure 1). Backward Recall of 5-digits saw significant improvement over time (*p* = 0.009), with pairwise differences from E2 to E3 (*p* = 0.009) and E2 to E4 (*p* = 0.002) (Figure 1A). Immediate 5-Word Recall for mTBI patients saw statistically significant change across time (*p* = 0.007) (Figure 1B). Single-Leg, Tandem-Leg, and Double-Leg Stances as well as total score of the modified BESS saw significant changes over all 4 encounters (*p* < 0.05 for all) (Figure 1C). While Tandem- and Double-Leg tests improved by less than one scoring item from E1 to E4, Single-Leg Stance had the largest change from E1 to E4 (4.0 errors to 1.6 errors, *p* < 0.001).

Multiple assessments were also able to distinguish mTBI subjects from controls. 5- and 6-Digit Backward Recall were significantly different than controls at all time points (*p* < 0.01 for all). Single-Leg Stance differentiated between mTBI cases and controls at each time point (*p* < 0.001 for all encounters). Double-Leg and Tandem-Leg Stances as well as total BESS scores were also significantly different than controls across encounters, but this difference was less than one scoring item.

Backward recall of 3- and 4-digits saw no significant change over time in mTBI patients, with most correctly recalling the digits 90% of the time or better.

### Demographic factors on outcomes over time

**Figure 2:**
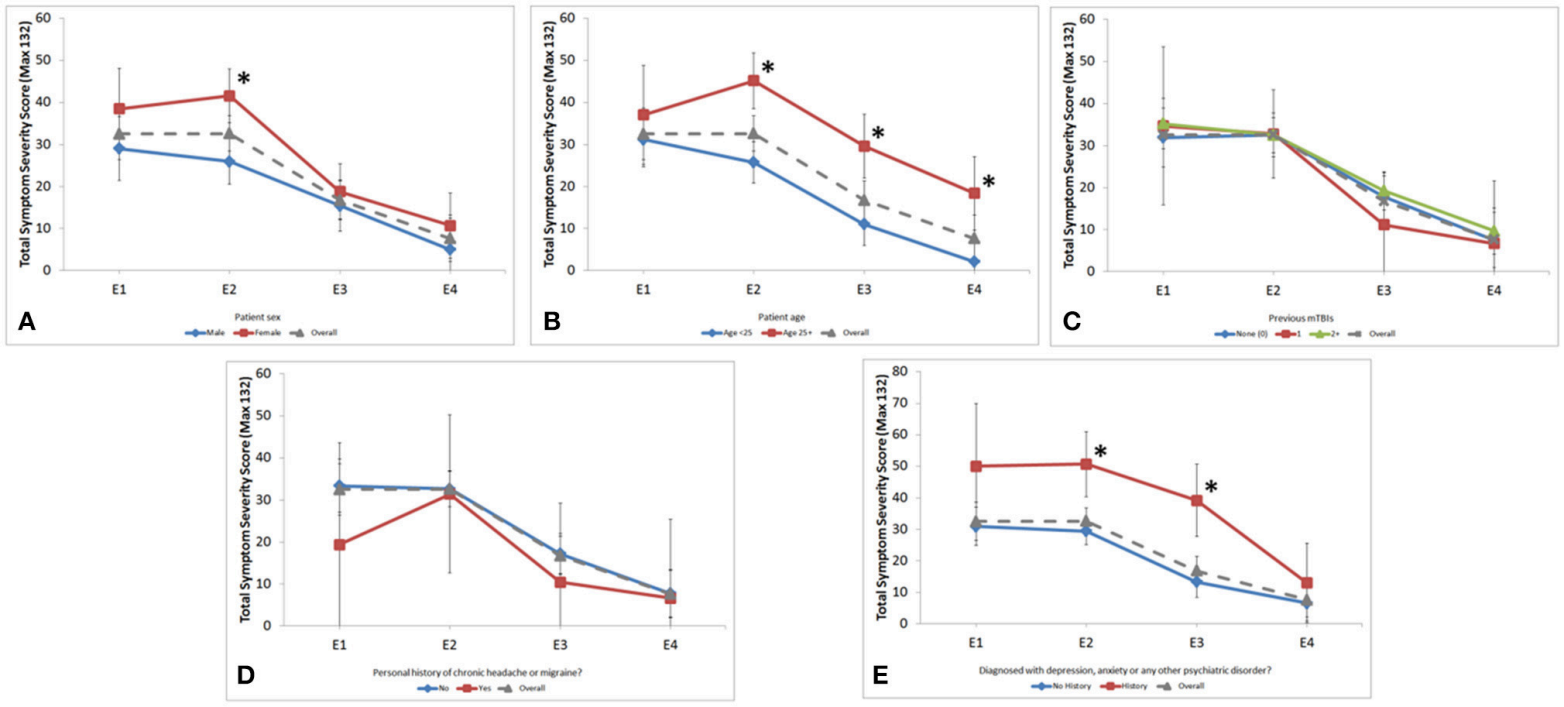
Longitudinal assessment of symptom severity score across encounters stratified by **(A)** sex; **(B)** age group; **(C)** number of previous mTBIs; **(D)** personal history of chronic headache or migraine and; **(E)** personal history of psychiatric disorder. Gender was significantly different at the E2 encounter (41.6 vs. 25.9, *p* < 0.001). Age was significantly different at E2 (46.0 vs. 28.0, *p* < 0.001), E3 (26.1 vs. 13.8, *p* < 0.001), and E4 (17.7 vs. 2.6, *p* = 0.003). Psychiatric history was significantly different at E2 (50.7 vs. 29.4, *p* < 0.001) and E3 (39.2 vs. 13.3, *p* < 0.001). ^*^p ≥ 0.05.

Multiple demographic factors were found to influence symptoms and recovery. Females reported higher symptoms scores at each time point with a significant difference at E2 (41.6 vs. 25.9, *p* < 0.001) (Figure 2A). Differences in clinical outcomes between sex can be found in Table 2. Patients aged ≥25 had higher symptom scores at E2 (*p* < 0.001), E3 (*p* < 0.001), and E4 (*p* = 0.003) (Figure 2B). Age also influenced the neuropsychological assessments. For subjects aged < 25, there was significant longitudinal improvement in 5-Digit Recall (*p* = 0.045), Immediate 5-Word Recall (*p* = 0.027), and BESS Single-Leg Stance (*p* = 0.023). Subjects aged ≥25 years did not significantly improve over time in any of these assessments.

**Table 2:** Comparisons of outcomes over time between gender.

**Variable**	**Encounter**	**Male**		**Female**		***P*-value (B)**
		**Mean**	**SE**	**Mean**	**SE**	
Symptom Severity Score (0–132)	E 1	29.0	3.9	38.4	4.9	0.134
	E 2	25.9	2.8	41.6	3.2	<0.001
	E 3	15.4	3.1	18.8	3.4	0.473
	E 4	5.0	3.8	10.7	4.0	0.303
	*P*-value (W)	<0.001		<0.001		
3-Digit Recall (% correct)	E 1	97%	3%	94%	5%	0.741
	E 2	98%	2%	95%	3%	0.425
	E 3	100%	0%	97%	3%	0.311
	E 4	97%	3%	96%	4%	0.960
	*P*-value (W)	0.360		0.944		
4-Digit Recall (% correct)	E 1	90%	6%	89%	7%	0.934
	E 2	93%	3%	83%	6%	0.155
	E 3	96%	3%	87%	5%	0.166
	E 4	93%	5%	93%	5%	0.943
	*P*-value (W)	0.713		0.630		
5-Digit Recall (% correct)	E 1	62%	9%	61%	11%	0.948
	E 2	48%	7%	51%	8%	0.770
	E 3	73%	7%	66%	8%	0.497
	E 4	70%	8%	78%	8%	0.502
	*P*-value (W)	0.005		0.100		
6-Digit Recall (% correct)	E 1	55%	9%	50%	12%	0.730
	E 2	53%	7%	41%	8%	0.270
	E 3	59%	7%	53%	8%	0.556
	E 4	60%	9%	52%	10%	0.535
	*P*-value (W)	0.813		0.681		
Immediate Memory Word Recall (1–15)	E 1	14.1	0.2	14.3	0.2	0.385
	E 2	14.4	0.1	14.4	0.2	0.730
	E 3	14.7	0.2	14.6	0.2	0.706
	E 4	14.5	0.2	14.9	0.2	0.177
	*P*-value (W)	0.050		0.117		
Total mBESS Score (0–30)	E 1	24.5	0.7	23.7	0.9	0.451
	E 2	25.8	0.5	26.5	0.6	0.392
	E 3	26.5	0.5	27.1	0.6	0.436
	E 4	27.4	0.7	28.0	0.7	0.527
	*P*-value (W)	0.014		0.001		
Double-Leg Stance (number of errors)	E 1	0.0	0.1	0.8	0.1	<0.001
	E 2	0.0	0.1	0.1	0.1	0.734
	E 3	0.0	0.1	0.1	0.1	0.637
	E 4	0.0	0.1	0.0	0.1	>0.999
	*P*-value (W)	0.978		<0.001		
Single-Leg Stance (number of errors)	E 1	4.0	0.4	3.9	0.5	0.936
	E 2	3.0	0.3	2.8	0.4	0.684
	E 3	1.9	0.3	1.9	0.4	0.874
	E 4	1.8	0.4	1.4	0.4	0.467
	*P*-value (W)	<0.001		0.001		
Tandem Stance (number of errors)	E 1	1.48	0.27	1.56	0.35	0.870
	E 2	1.15	0.20	0.83	0.23	0.303
	E 3	0.89	0.22	0.92	0.24	0.916
	E 4	0.77	0.27	0.68	0.28	0.821
	*P*-value (W)	0.227		0.240		

*B, indicates adjusted p-value between males and females; W, indicates overall p-value within males and females*.

Patients who reported personal history of depression, anxiety, or other psychiatric disorder had higher SSS at each time point, which was statistically significant at E2 and E3 (*p* = 0.003, *p* < 0.001 respectively; Figure 2E). This finding was not influenced by sex or age.

Number of previous mTBIs, learning disabilities, and personal history of chronic headache/migraine did not correlate with SSS or longitudinal change of SSS (Figures 2C,D).

## Discussion

This prospective, longitudinal study compared history, symptoms, and imaging results in a group of patients with mild TBI and non-injured controls. The acute enrollment post-trauma followed by multiple encounters over 3 months provides robust data that allows preliminary conclusions to be drawn regarding clinical symptoms, recovery, risk factors, and the usefulness of neurological assessments and MRI. To date, this is one of the first studies of this size to longitudinally assesses both clinical neurological exams and MRI data, in a design that captures more than 3 time points (24, 25). Given the young and otherwise healthy patient population, there were limited confounds in post-concussive deficits from mild cognitive impairment, other medical illness, or dementia that have historically impacted earlier mTBI studies. The demographic distribution allowed for sex- and age-stratified analyses that are not present in most other studies.

### Symptoms

mTBI symptoms are not universal and vary tremendously between patients. However, the quantitative symptom severity score is considered a reliable approximation of the severity of a patient's injury and has previously been shown to predict prolonged recovery given that concussion currently remains a symptom-based diagnosis (26, 27). Although previous studies and meta-analyses have determined that neuropsychological deficits typically recover within 3 months, subjective report of difficulty remembering was the most frequently cited symptom at 3 months post-injury in our mTBI study cohort (17, 28). Additionally, both symptoms and neuropsychological deficits in our population were present longer than in previously cited literature (15, 16, 29). We suggest that future studies should more carefully consider longer term, subtle cognitive deficits in this population.

### Structural imaging

White matter abnormalities were seen in a larger percent of this mTBI cohort than previously documented in normal, healthy populations (27 vs. 5.3% in adults and 1.2% in adolescents) (30, 31). Direct comparison of findings however, are limited by differences in MR technologies. Due to greater signal to noise ratio, modern high-field volumetric T2 FLAIR imaging is significantly more sensitive in detecting white matter foci than reported in the historical literature (32), which may contribute to these slightly higher rates. The incidence rates of white matter abnormalities reported in the current study cannot be explained by hypertension, diabetes, demyelinating diseases as neither comorbidity was present in this study population, which was predominantly under the age of 30. Additional larger, prospective studies are needed to clarify the relationship between white matter hyperintensities and acute mTBI.

### Assessments

Although both 5- and 6-Digit Backward Recall differentiated between mTBI cases and controls, only 5-Digit Backward Recall was useful in evaluating mTBI patients across the first 3 months post-injury because of the noticeable longitudinal trend demonstrating improvement. mTBI patients appear to struggle with 6-Digit Backward Recall across all encounters compared to controls, suggesting that this test is helpful in diagnosing mTBI but is less helpful in tracking cognitive improvement over time. 3- and 4-digit recall may have less utility than 5- or 6-digit recall when assessing mTBI patients.

Clinicians continue to employ a simple 3-Word Recall test, dated from 1975, that does not appear to capture the memory deficit in the acute mTBI patient (12). How many words then are needed? The SCAT test has undergone multiple iterations, all of which include 5-Word Recall (10, 33). Although our research has demonstrated Immediate 5-Word Recall to be statistically significant over time, this test may not achieve the desired clinical sensitivity given the average change of one word. Clinicians may considering employing increasing numbers of words in order to better assess the memory deficit in acute mTBI.

Although all three portions of the Modified BESS assessment demonstrate significant improvement over time, the Single-Leg Stance appears to be the most sensitive in the acute window and the most advantageous position of this composite given its proportional decrease in scores across encounters.

### Demographic risk factors

Sex influenced symptom severity and course of recovery, with females experiencing worse symptoms at each time point, consistent with previously published literature (34–37). Studies cite that women exhibit higher rates of cognitive impairment than men after an mTBI (38). This was not captured in the assessments employed in this pilot study, suggesting a need for more sophisticated neuropsychological testing in future studies.

Age may also be a variable in mTBI severity and recovery. Data were analyzed for subjects ≥ and < 25 years, as 25 is the age when most brain structures have reached maturity (21–23). Our results show that individuals aged ≥25 endorse more symptoms after their first visit and generally take longer to recover. This is also seen in the significantly older age of the non-recovered cohort of patients. Still, the study cohort was youthful, as 80% of subjects were aged <30. These results are contrary to previous findings in the 13 to 33 age range that have shown younger age to be a possible risk factor for more severe neuropsychological deficits and longer recovery (39, 40). However, the majority of the recent mTBI literature in similar age groups focuses on sport populations. It is possible that the establishment and reinforcement of the neurocircuitry and pathways that occur between the ages of 15–25 may exert a protective effect, which remains poorly understood and needs to be addressed in future studies (41).

Studies conflict in terms of the relevance of personal psychiatric or headache history on prognosis in mTBI (13). It is interesting to note that a personal psychiatric history led to significantly worse symptoms acutely but did not independently predict recovery of patients in this study.

Recent advancements in TBI serum biomarkers have resulted in Federal Drug Administration (FDA) approval for the use of blood tests in the evaluation of mTBI (42). However, the evaluation of blood-based biomarkers was not within the scope of the current study.

### Limitations

Our data was meant to be hypothesis generating by reporting observations, limiting statistical power analyses. Although steps were taken to limit differentiation between institutions, there may have been slight residual instrumental variability between the enrollment sites despite standardization efforts. Our study was limited to neurocognitive assessments that are not normalized by age, although we controlled for age in our linear regression models. It is unclear if the non-significant findings of sex, age, and psychiatric history at E1 were due to timing of entry into the study at E1 or statistical power. Lastly, attrition limited longitudinal data analysis.

## Conclusion

This study was meant to provide *a priori* hypotheses for future studies. The findings can help inform clinicians in their post-TBI neurologic exam and draw attention to specific risk factors for prolonged recovery. Our findings suggest that female sex is a risk factor for increased symptom burden. Similarly, age greater than 25, history of depression, anxiety, and other psychiatric disorders were associated with greater symptom severity over time. Based on our findings, we hypothesize that sex, age, and psychiatric comorbidity may influence the course of recovery from mild TBI and should be further investigated. We also hypothesize that the observed higher prevalence of white matter hyperintensities in our mTBI and control subjects is due to MR technological advancements. Further validation with larger populations of subjects is needed for all these findings. Differences in clinical neurological exams between mTBI patients and controls demonstrate valid use of our tools for the study of future mTBI neuroimaging. It is also important to note that in light of recent advancements of TBI serum biomarkers, it is critical that clinical data in tandem with both blood-based and imaging biomarkers of mTBI may lead clinicians toward a more definitive model of care.

## Ethics statement

This study was carried out in accordance with the recommendations of the Hospital for Special Surgery, Houston Methodist, and University of California San Francisco Institutional Review Boards. The protocol was approved by the Hospital for Special Surgery, Houston Methodist, and University of California San Francisco Institutional Review Boards. All subjects gave written informed consent in accordance with the Declaration of Helsinki. All minors signed an additional assent form before participating.

## Author contributions

TS obtaining funding, study concept and design, study supervision, acquisition of data, interpretation of the data, drafting the manuscript. JN analysis and interpretation of data, drafting the manuscript. TC, AD, KH, EK, and KC interpretation of the data, drafting the manuscript. AT acquisition of data, drafting the manuscript. SN acquisition of data, drafting the manuscript for intellectual content. TZ analysis and interpretation of data, drafting of manuscript for intellectual content. JM and PM study concept and design, acquisition of data, study supervision, revising the manuscript for intellectual content. LM study concept and design, study supervision, processing of data, analysis and interpretation of data, drafting the manuscript.

### Conflict of interest statement

TS has grants from GE and the NFL, Chembio Diagnostics, ElMindA, and Teva Pharmaceuticals. She is a member of the GE NFL Medical Advisory Board. JN has received grant support from the National Center for Advancing Translational Services (NCATS). TZ and LM are employed by General Electric. JM and PM have a grant from GE and the NFL and are members of the GE NFL Medical Advisory Board. The remaining authors declare that the research was conducted in the absence of any commercial or financial relationships that could be construed as a potential conflict of interest.

## Abbreviations

AUC: area under the curve
BESS: balance error scoring system
CGSA: composite general symptoms assessment
E1: encounter 1
E2: encounter 2
E3: encounter 3
E4: encounter 4
FLAIR: fluid attenuation inversion recovery
GCS: Glasgow coma scale
HIPPA: Health Insurance Portability and Accountability Act
HM: Houston Methodist
HSS: Hospital for Special Surgery
MRI: magnetic resonance imaging
mTBI: mild traumatic brain injury
RF: Radiofrequency
SCAT2: sports concussion assessment tool 2nd edition
SSS: symptom severity score
TBI: traumatic brain injury
UCSF: University of California, San Francisco.
