# Supplementary material for: Clinical Findings in a Multicenter MRI Study of Mild TBI
Source: Front Neurol. 2018 Oct 23;9:836. doi: 10.3389/fneur.2018.00836 (PMC6206843; doi:10.3389/fneur.2018.00836)
Supplement: Supplementary file 1 [file Data_Sheet_1.DOCX]

**Appendix e-1: Inclusion and Exclusion Criteria**

Patients were excluded from enrollment for (1) loss of consciousness greater than 15 minutes after injury, (2) posttraumatic amnesia lasting greater than 24 hours after TBI event, (3) scoring less than 13 on the Glasgow Coma Scale (GCS), (4) a structural brain injury indicated by previous neurological findings, (5) a previous history of moderate or severe TBI, (6) another mTBI within 12 months, (7) a brain white matter disease, (8) history of seizures within past 10 years, (9) history of self-reported recreational drug usage in past 10 years, (10) history of alcohol abuse or dependence (per DSM-IV-TR Diagnostic Criteria), (11) current primary Axis I or II psychiatric disorder, except for disorders classified as minor and not expected to impact study conduct or integrity (12) history of brain mass, neurosurgery, stroke, or dementia, (13) known cognitive dysfunction, structural brain disease, or malformation, or (14) current anti-psychotic, psychotropic, or antiepileptic medication usage.
