# Supplementary material for: Clinical Findings in a Multicenter MRI Study of Mild TBI
Source: Front Neurol. 2018 Oct 23;9:836. doi: 10.3389/fneur.2018.00836 (PMC6206843; doi:10.3389/fneur.2018.00836)
Supplement: Supplementary file 2 [file Data_Sheet_2.DOCX]

**Appendix e-2: Description of GEHC Composite General Symptoms Assessment**

The CGSA was compiled as a comprehensive compilation of the Sports Concussion Assessment Tools 2 and 3 (SCAT2 and SCAT3), Sports Concussion Office Assessment Tool (SCOAT), Glasgow Coma Scale (GCS), Extended Glasgow Outcome Scale (GOS-E), NFL Sideline Assessment tool, and Concussion Graded Symptoms Checklist (GSC). Notably, the tool contains the validated modified Balance Error Scoring System (BESS) and Sports Assessment of Concussion (SAC) tools, which are found as part of the SCAT 2, SCAT 3, SCOAT, and NFL Sideline Assessment Tools.

The symptoms collected were as follows: Anxious, Balance problems, Blurred Vision, Confusion, Depressed, Difficulty concentrating, Difficulty remembering, Dizziness, Don’t feel right, Drowsiness, Easily Distracted, Fatigue, Low Energy, Feeling in a Fog, Feeling Slowed Down, Headache, Irritability, Loss of Consciousness, Loss of Orientation, Memory Problems, Nauseous, Neck Pain, Nervousness, Numbness, Personality Changes, Photo-phobia, Phono-phobia, Poor Balance/Coordination, Pressure in Head, Ringing in the Ears, Sadness, Seeing Stars, Sensitivity to Light, Sensitivity to Noise, Sleep – Difficulty falling asleep, Sleep Disturbances, Sleeping More than Usual, Sleeping Less than Usual, Tingling, Trouble Falling Asleep, Unusually Emotional, Vacant Stares/Glass Eyes, Visual Problems, and Vomiting.

The assessments completed were: Balance examination (modified BESS of single-leg stance, tandem stance, and double-leg stance), Extended Glasgow Outcome Scale (GOS-E), Glasgow Coma Scale (GCS) and Pupil Information, neck examination, cognitive assessments (date, year, time, etc.), 5-Word Recall, Digits Backward (3-6 digits), Months Backward, Coordination Examination,

The comorbidities collected were: chronic headaches, migraine, epilepsy, thyroid dysfunction, encephalitis, meningitis, developmental history of learning disability or attention deficit, anxiety, depression, and sleep disorders.
