# Supplementary material for: Clinical Findings in a Multicenter MRI Study of Mild TBI
Source: Front Neurol. 2018 Oct 23;9:836. doi: 10.3389/fneur.2018.00836 (PMC6206843; doi:10.3389/fneur.2018.00836)
Supplement: Supplementary file 3 [file Data_Sheet_3.DOCX]

**Appendix e-3: Imaging Protocol**

MR imaging was performed on 3T GE Signa MR750 (GE Healthcare, Waukesha, WI) scanners with a 32-channel brain radiofrequency coil (Nova Medical, Wilmington, MA). The MR imaging protocol included: sagittal MP-RAGE 3D T1 (matrix: 284x284x180, field-of-view = 25.6cm, slice thickness = 0.9mm, flip angle = 8 degrees, bandwith = 31.25 kHz, TR/TE/TI = 8.1ms / 3.3ms (Min Full) / 1,100ms, ARC acceleration = 2 (phase) x 1.5 (slice), scan time = 4:33); sagittal 3D T2 FLAIR (matrix: 256x256x164, field-of-view = 25.6cm, slice thickness = 1.0mm, bandwidth = 31.25kHz, echo train length = 200, TE/TR = 119ms / 6800ms, ARC acceleration = 2 (phase) x 2 (slice), scan time = 5:50) ; axial diffusion MRI (single spin echo, matrix: 96x96x21, field of view = 24cm, slice thickness = 2.5mm, bandwidth = 250 kHz, flip angle = 90 degrees, TR/TE = 2,600ms / ~77ms (Min TE), ARC acceleration = 2 (phase), simultaneous multi slice acceleration factor = 3, 140 directions distributed on three shells – 25, 40, 75 directions per shell – with b-values 800, 1200, 2800 mm^2^/s with 7 interspersed T2 volumes, scan time = 6:56); axial multi-echo SWAN (matrix: 480x480x76, slice thickness = 2.0mm, bandwidth = 62.50kHz, Min TR, TE=5.4ms, 11.0ms, 16.5ms, 22.1ms, 27.7ms, 33.3ms (6 echoes), flip angle =15 degrees, ASSET acceleration = 2 (phase), scan time = 6:29).
